# Editorial: The Function of Phagocytes in Non-Mammals

**DOI:** 10.3389/fimmu.2020.628847

**Published:** 2020-12-11

**Authors:** Xin-Jiang Lu, Qing Deng, Kim Dawn Thompson

**Affiliations:** ^1^ State Key Laboratory for Managing Biotic and Chemical Threats to the Quality and Safety of Agro-products, Ningbo University, Ningbo, China; ^2^ Laboratory of Biochemistry and Molecular Biology, School of Marine Sciences, Ningbo University, Ningbo, China; ^3^ Key Laboratory of Applied Marine Biotechnology of Ministry of Education, Ningbo University, Ningbo, China; ^4^ Laboratory for Marine Biology and Biotechnology, Pilot National Laboratory for Marine Science and Technology, Qingdao, China; ^5^ Department of Biological Sciences, Purdue University, West Lafayette, IN, United States; ^6^ Aquaculture Research Group, Moredun Research Institute, Penicuik, United Kingdom

**Keywords:** phagocytes, non-mammals, macrophages, neutrophils, B cells, macrophage polarization, hemocytes, teleost

Non-mammalian animals are useful models for understanding the evolution of the immune system and studying the underlying mechanisms of cellular and molecular responses in mammalian immunity. Recent literature has expanded the function of phagocytes beyond pathogen killing to tissue-supporting activities (1). In humans, many diseases that stem from dysregulated phagocyte function, such as congenital neutropenia, familial Mediterranean fever, and sepsis (2–4), still lack satisfactory treatment regimens. Because of the high degree of conservation present in basic functions of the innate immune system, it is our opinion that research on non-mammalian phagocytes will provide novel insight into new paradigms for mammalian phagocyte counterparts. For example, extracellular chromatin from phagocytes participates in host defenses found in both invertebrates and vertebrates (5). Indeed, the use of zebrafish models has advanced our understanding of phagocyte functions, in human tuberculosis, for example (6). The composition of phagocytes in non-mammals is more diverse. Phagocytes in mammals consist mainly of macrophages and neutrophils, while in teleosts, B lymphocytes and thrombocytes from peripheral blood also have potent phagocytic activity (7, 8). Teleost-specific genome duplication events produce genes with overlapping functions, potentially resulting in sub-functionalization in the teleost immune system. An example of this is CXCR3.1 and CXCR3.2 in teleosts, which differentially contribute to macrophage polarization (9).

The collection of 14 papers within this Research Topic contributes substantially to the development of research tools and improved understanding of the function and regulation of phagocytes in non-mammals. Wu et al. reviewed recent advances of phagocytic B cells and their microbicidal ability in teleosts. Experimental evidence demonstrated that in both teleosts and mammals, phagocytic B cells could recognize, take up, and destroy particulate antigens and then present those processed antigens to CD4^+^ T cells to elicit adaptive immune responses. Future prospective studies will focus on the fundamental differences in phagocytic processes between B cells and classical phagocytes, such as macrophages and neutrophils. Linnerz et al. reviewed new findings of underlying cellular and molecular mechanisms for a variety of phagocyte responses in zebrafish. They also described signaling pathways that controlled the recruitment and fate of phagocytes at inflammatory sites of zebrafish. Furthermore, they demonstrated the limitations and opportunities for studying phagocytes in zebrafish, which found early embryonic and larval stages to be a good model for studying the innate immune response without interference of adaptive immunity. Sommer et al. reviewed the role of two main classes of chemokine receptors, the CC and CXC subtypes, in phagocyte migration in zebrafish models for cancer, infectious disease, and inflammation, with special emphasis on chemokine receptors and atypical chemokine receptors in shaping self-generated chemokine gradients. In addition, they used the functional antagonism between two paralogs of the CXCR3 family as an example to illustrate divergence and sub-functionalization of chemokine receptors in zebrafish.

Wentzel et al. described the metabolic signatures associated with macrophage polarization. They found decreased oxidative phosphorylation and increased glycolysis in M1 macrophages, while similar oxidative phosphorylation and glycolysis were found between M2 and control macrophages. Their results suggest that immunometabolic reprogramming determined the inflammatory phenotype of polarized macrophages in teleost fish such as carp, similar to what happens in mammals. Ultimately, this helps to improve our understanding of the fundamental mechanisms underlying energy metabolism and metabolic reprogramming of immune cells in teleost fish.

Liu S. et al. reviewed the current knowledge of hemocyte phagocytosis in crustaceans. The authors summarized phagocytosis related receptors for recognition and internalization of pathogens, as well as the downstream signal pathways and intracellular regulators involved in phagocytosis in crustaceans. Different subpopulation of hemocytes in diverse species of crustaceans exhibited variable phagocytic activities. Further investigation is necessary to reveal phagosome formation and maturation, as well as microbe destruction in the hemocytes of crustaceans. Liu C. et al. identified CD63 as a receptor that participates in immune recognition and hemocyte phagocytosis in oyster, and found CD63 to be recruited to the phagosomes after *Yarrowia lipolytica* stimulation. The CD63 receptor showed binding capacity to glucan, peptidoglycan, and lipopolysaccharide. Their work indicates that CD63 may function as a gateway between the pattern recognition of foreign invaders and the immune response of oysters. Lin et al. established a facile magnetic cell sorting method to enrich professional phagocytes from hemocytes of Hong Kong oyster. Pathway annotation of phagocytic processes of these hemocytes revealed that focal adhesion and extracellular matrix–receptor interactions were the most conspicuously enriched pathways in phagocytes by transcriptomic analysis. Focal adhesion kinase and heparan sulfate proteoglycans were identified as major regulators in anti-bacterial defense of hemocytes. Mao et al. isolated granulocytes and hyalinocytes by flow cytometry from Pacific oyster hemocytes. Cdc42 was identified as a core regulator of phagocytic pathways in these cells through an array of differentially expressed genes. The AP-1 transcription factor Fos was confirmed to facilitate functional differentiation of hemocytes in an assay on binding to target genes by the AP-1 binding site. Their works provide mechanistic underpinnings of functional differentiation of hemocytes in a marine invertebrate Pacific oyster.

Yaparla et al. investigated the production of phagocytes in amphibians. They confirmed that while the liver periphery of *Xenopus laevis* hosts hematopoietic stem cells and megakaryocyte/erythroid potentials, their bone marrow contains granulocyte/macrophage potentials. They also demonstrated that condition medium from *X. laevis* bone marrow cells chemo-attracts liver periphery and peripheral blood cells, supporting the concept that cells bearing granulocyte/macrophage potentials originate from the frog liver periphery. Sreekantapuram et al. established an *ex vivo* chicken whole-blood infection assay to analyze interactions between host cells and several model pathogens. Monocytes, and to a lesser extent heterophils, were shown to be associated with pathogens. They provide the first insight into quantitative interactions of three model pathogens with different immune cell populations in avian blood, demonstrating a broad spectrum of different characteristics during the immune response that depends on the pathogen and the chicken line. More-Bayona et al. evaluated the impact of xenobiotic mixtures on chicken immunity. An increase in resident macrophages and a decrease in CD8^+^ lymphocytes were observed in the abdominal cavity after acute exposure to contaminated water. Leukocyte recruitment into the challenge site and activation of phagocyte antimicrobial responses were also affected. They emphasize that it is necessary to consider the impact of xenobiotic mixtures in the assessments of water quality.

Soto-Dávila et al. evaluated the effects of Vitamins D2 and D3 on the phagocytic response of Atlantic salmon (*Salmo salar* L.) primary macrophage cultures to *Aeromonas salmonicida*. Vitamin D is an essential nutrient for finfish aquafeeds, although its role in fish cell immunity is unknown. The authors found vitamin D3 to induce anti-bacterial innate immunity pathways in macrophages, affecting bacterial attachment, infection, and growth within the macrophage cultures. Vitamin D2, on the other hand, had no effect. Pérez-Stuardo et al. treated Atlantic salmon macrophage-enriched cell cultures, infected with *Piscirickettsia salmonis*, with non-specific IgM-enriched beads and evaluated the effect that this treatment had on lysosomal (pH change) and proteolytic activity. Treatment with the IgM-beads reversed the modulation of lysosomal activity induced by the bacterial infection and promoted macrophage survival and bacterial elimination. Park et al. highlighted the usefulness of imaging flow cytometry (IFC) in phagocyte research—a technique combining flow cytometry with digital microscopy to generate quantitative high-throughput imaging data. The authors used IFC to study phagocytic cells from Atlantic salmon, Nile tilapia (*Oreochromis niloticus*), and blue mussel (*Mytilus edulis*), including the effects of incubation temperature on their ability to phagocytose degradable particles *in vitro*. While phagocytosis by fish phagocytes was affected by the incubation temperature, no effect of temperature was noted on the activity of blue mussel phagocytes.

Overall, the content of this Research Topic reflects the significant growth and advances in the field of phagocytes in non-mammals. It contributes to improved mechanistic understanding, which is interrelated with evaluating both cellular and molecular biological approaches to investigate the function, mechanism, and production of phagocytes. Further characterization of phagocytes in non-mammals will be focused on more detail mechanisms and new methods, which is invaluable to understand the evolution of phagocytes.

## Author Contributions

All authors listed have made a substantial, direct, and intellectual contribution to the work and approved it for publication.

## Funding

This project was supported by the Program for the Natural Science Foundation of China (41776151; 31972821), the Zhejiang Provincial Natural Science Foundation of China (LR18C040001; LZ18C190001), the Fundamental Research Funds for the Provincial Universities of Zhejiang (SJLZ2020002), and the National Institutes of Health (Grant R35GM119787 to QD).

## Conflict of Interest

The authors declare that the research was conducted in the absence of any commercial or financial relationships that could be construed as a potential conflict of interest.
